# Porcine interferon-alpha but not interferon-lambda inhibits replication of genotypically diverse PRRSV isolates *in vitro*

**DOI:** 10.3389/fvets.2026.1873208

**Published:** 2026-09-03

**Authors:** Samantha J. Hau, Steven G. Kellner, Nickolas Knudsen, Lillian Skidmore, Peter C. Canning, Susan L. Brockmeier

**Affiliations:** 1 National Animal Disease Center, ARS, USDA, Ames, IA, United States; 2 Ambrx, Inc., La Jolla, CA, United States; 3 Elanco Animal Health, Greenfield, IN, United States

**Keywords:** antiviral, interferon, porcine reproductive and respiratory syndrome virus, swine, treatment

## Abstract

**Introduction:**

Porcine reproductive and respiratory syndrome (PRRS) is an economically devastating viral infection in pigs that results in estimated losses of $1.2 billion annually. Control of porcine reproductive and respiratory syndrome virus (PRRSV) has been challenging due to the weak and delayed development of adaptive immunity to the virus. This has been partially attributed to the low induction of host interferon (IFN). IFNs are cytokines that induce an antiviral state in target cells and promote the development of the adaptive immune response to the virus. The use of exogenous IFNs are of interest in the control of PRRSV in the swine industry.

**Methods:**

The efficacy of porcine IFN-alpha (pIFNα) and porcine IFN-lambda (pIFNλ) at inhibiting PRRSV replication in porcine alveolar macrophages *in vitro* using a genotypically diverse subset of PRRSV isolates. Additionally, because the utility of many cytokine products *in vivo* is limited by half-life and dosing interval, PEGylated constructs of pIFNα were assessed for their capacity to inhibit PRRSV replication.

**Results:**

Porcine IFNα more efficiently inhibited PRRSV replication and induced greater transcription of IFN-stimulated genes than pIFNλ; however, the efficacy of PRRSV inhibition was strain dependent. PEGylated pIFNα also reduced PRRSV replication in pretreated cells; however, the efficiency of inhibition was reduced compared to wild type pIFNα.

**Discussion:**

Porcine IFNα was shown to be more capable of inhibiting PRRSV replication; however, some strains have reduced susceptibility to pIFN treatment. This work indicates PEGylated pIFNα retains functional activity and has potential as a biotherapeutic product to control PRRSV replication in pigs.

## 1 Introduction

Interferons (IFNs) are a family of cytokines that play an important role in innate and adaptive immunity. They can alter the course of viral infection by inducing an antiviral state in cells that limits viral replication and promotes immune surveillance and killing of infected cells (1). They can also impact the virus specific immune response by altering the helper T cell phenotype and cytokine production resulting in greater TH1 skew (2, 3). IFNs are divided into three classes based on their receptor complex (4), Type I-III. Type I and Type III IFN display similar antiviral activity in target cells and both show efficacy as biotherapeutic products to control viral infections in humans (4, 5).

Porcine reproductive and respiratory syndrome virus (PRRSV) is a globally disseminated pathogen of swine causing reproductive losses and pneumonia resulting in annual losses estimated at $1.2 billion (6). Infection with PRRSV does not result in a strong IFN response (7), which may contribute to delayed development of adaptive immunity and delayed viral clearance (8, 9). IFNs have been evaluated *in vitro* and *in vivo* for their effects on PRRSV replication, clinical disease, and adaptive immune responses (7, 10–20). The use of IFN-alpha in pigs has been shown to limit viral replication, delay peak viral titers, and reduce clinical disease in PRRSV infected animals (13–16); however, the IFN-alpha mediated effects were limited. In the case of an adenovirus-vectored IFN-alpha, limited efficacy may be due to a rapid decline of IFN levels following administration, with pigs returning to baseline levels by 4 days post administration (14). One strategy to combat the rapid decline of IFN activity is conjugating IFN to polyethylene glycol (PEGylation), which causes increased drug stability and prolonged half-life (21).

In this report, we assessed both porcine IFN-alpha (pIFNα) and porcine IFN-lambda (pIFNλ) for ability to suppress viral replication in a genomically diverse group of PRRSV isolates and induce IFN-stimulated gene transcription in porcine alveolar macrophages. pIFN-alpha was better able to inhibit viral replication and better induced IFN-stimulated genes *in vitro*. Additionally, because modification of cytokines can result in reduced biological activity (21), we evaluated the effects of PEGylation on the ability of pIFNα to suppress PRRSV replication. pIFNα PEGylated constructs were able to decrease viral replication, though at a reduced capacity compared with the wild-type pIFNα.

## 2 Materials and methods

### 2.1 PRRSV strains

Nine PRRSV strains were utilized in this study SDSU73, MN-184, NADC-8, NADC-20, NADC-30, VR2332, SRV07, JXwn06, and Lelystad (22–31). Strains were selected to be a broad representation of PRRSV genotypes, with type 1, type 2, and high pathogenicity isolates represented. Isolate information can be found in Supplementary Table S1.

### 2.2 Generation of recombinant pIFNs

Recombinant pIFNα and pIFNλ1 were produced by Ambrx, Inc. (San Diego, CA) using an *Escherichia coli* expression system. Both proteins were expressed as inclusion bodies, isolated, refolded, and purified to homogeneity. Following purification, pIFNα was formulated in 270 mM glycine and 4% trehalose at pH 4.0, while pIFNλ1 was formulated in 20 mM sodium citrate and 150 mM NaCl at pH 6.5 with final endotoxin levels reading below 2.0 EU/mg. Final molecules were 0.22 μm filtered, frozen, and stored at −80 °C until use.

### 2.3 Generation of PEGylated porcine IFNα

Recombinant PEGylated pIFNα was generated using the Ambrx *E. coli* expression system engineered for site-specific incorporation of synthetic amino acids into protein sequences. Using this system, pIFNα was expressed with synthetic amino acid, p-acetyl-L-phenylalanine (pAcF), incorporated at one of the following amino acid sites to facilitate site-specific PEGylation: E103, E107, or L112. These sites were selected as preferred PEGylation sites due to improved antiviral activity, biophysical characteristics, and pharmacokinetic profile compared to other site variants. The recombinant pIFNα variants, expressed into inclusion bodies, were isolated, refolded, and purified to homogeneity. Following purification, pIFNα variants were site-specifically conjugated at the pAcF site with a single aminooxy functionalized 30 kDa polyethylene glycol (PEG) molecule through a stable oxime bond. PEGylated pIFNα constructs were further purified to remove excess reagents from the conjugation reaction and formulated in 20 mM sodium acetate, 150 mM NaCl, 5% glycerol, and 0.005% Tween 80 with final endotoxin levels reading below 2.0 EU/mg. Final molecules were 0.22 μm filtered, frozen, and stored at −80 °C until use.

### 2.4 Porcine alveolar macrophage collection

Porcine pulmonary alveolar macrophages (PAMs) were collected as described previously from young, healthy mixed-breed animals (32, 33). Briefly, the lungs of animals were repeatedly flushed with PBS containing Penicillin–Streptomycin (Sigma-Aldrich) to obtain approximately 750 mL of fluid. Lavage fluid was centrifuged at 400× g for 15 min, cells were washed with PBS and resuspended in RPMI supplemented with 5% fetal bovine serum, 5 mM HEPES, 1 mM L-glutamine, and Penicillin–Streptomycin. Cells were cultured in 150 × 15-mm petri dishes for 2 h at 37 °C with 5% CO_2_. Following incubation, media and nonadherent cells were aspirated and adherent cells rinsed with PBS. Adherent cells were harvested in PBS with a cell scraper, collected by centrifugation, washed once with PBS, resuspended in RPMI, and quantified using a hemocytometer. PAMs were seeded into 48-well plates in 500 μL volumes containing 5 × 10^5^ cells and allowed to adhere for 1 h.

### 2.5 Experimental design

#### 2.5.1 Evaluation of recombinant pIFNα and pIFNλ1 titrations in preventing PRRSV replication in PAMs

The recombinant pIFNs were titrated using two biological replicates against SDSU73 in PAMs isolated from two different animals. Adhered PAMs were exposed to RPMI containing dilutions of pIFNα or pIFNλ1 (10 ng/mL, 100 ng/mL, or 1,000 ng/mL) or RPMI without IFN for 16 h prior to infection. After IFN exposure, PAMs were infected with SDSU73 in cell culture media at an MOI of 1, as quantified using TCID_50_, for 2 h. After 2 h, PAMs were washed and RPMI with IFN at the previous concentrations was added to wells. Non-infected wells served as a control. Supernatants were collected for PRRSV quantification at 12, 24, 36, and 48 h post-infection (hpi). Supernatants were serially diluted and frozen at −80 °C until viral isolation and quantification.

#### 2.5.2 Efficacy of pIFNα and pIFNλ1 against infection with diverse PRRSV isolates in PAMs

Recombinant pIFNs were assessed for their efficacy against eight PRRSV isolates using two biological replicates with PAMs isolated from two different animals. Adhered PAMs were exposed to RPMI containing 1,000 ng/mL of pIFNα or pIFNλ1 or without IFN for 16 h prior to infection. Media containing IFN was removed and PAMs were infected with MN-184, NADC-8, NADC-20, NADC-30, VR2332, SRV07, Jxw06, or Lelystad at an MOI of 1 for 2 h. After PRRSV infection, PAMs were washed and RPMI with or without IFN as prior to infection was added to the wells. Control wells included non-treated/infected, treated/non-infected, and non-treated/non-infected Supernatants were collected at 12, 24, 36, and 48 hpi, serially diluted, and frozen at −80 °C until viral isolation and quantification.

#### 2.5.3 Efficacy of pIFNα and pIFNλ1 against PRRSV infection in MARC-145 cells

MARC-145 cells were seeded in 24-well plates and allowed to grow to confluence. Prior to infection, cells were exposed to 1,000 ng/mL pIFNα or pIFNλ1 or no IFN for 16 h. Wells were infected with SDSU73 at an MOI of 1 for 2 h. Following infection, wells were washed and media with or without IFN was reapplied. Supernatant was collected at 12, 24, 36, and 48 hpi, serially diluted, and stored at −80 °C until viral isolation and quantification. Assessment of pIFN efficacy in MARC-145 cells was done using two technical replicates.

#### 2.5.4 Assessment of induction of IFN stimulated genes (ISGs) in PAMs and PK-15 cells

PAMs from two pigs and PK-15 cells were exposed to cell culture media containing 1,000 ng/mL of pIFNα, pIFNλ1, or no IFN as described during infection assessment. Cells were mock infected for 2 h, washed, and incubated with IFN containing media or media without IFN. Cells were collected after 4, 8, 12, and 24 h in TRIzol reagent (Thermo Fisher Scientific) following the manufacturer’s recommendations and stored at −80 °C until RNA extraction.

#### 2.5.5 Assessment of pIFNα constructs in MARC-145 cells

PEGylated IFNα constructs (E103, E107, L112) and non-PEGylated wild type (WT) IFN were titrated (100 ng/mL, 500 ng/mL, and 1,000 ng/mL) and assessed for efficacy preventing PRRSV replication. MARC-145 cells were seeded in 24-well plates and grown to confluence. Media was removed and replaced with media containing a PEGylated IFN titration or control media with no IFN for 16 h prior to infection. MARC-145 cells was exposed to SDSU73 in cell culture media at an MOI of 1 for 2 h. After incubation, cells were washed and media containing PEGylated IFN or control media was re-applied to the cells. Supernatants were collected at 24, 48, 72, and 96 h post-infection to assess PRRSV replication. Ten-fold dilutions of the supernatants were aliquoted and frozen at −80 °C for future analysis. Controls included non-infected, non-challenged wells and non-infected, challenged wells at each time point.

### 2.6 PRRSV isolation and quantification

PRRSV infection was assessed using virus isolation and titration in MARC-145 cells and real-time, reverse transcriptase PCR (RT-qPCR). Virus isolation was performed on cell supernatants. MARC-145 cells in 24-well plates were exposed to 100 μL of cell supernatant from test wells at the assessed time points. After incubating for 2 h, test supernatant was replaced with fresh cell culture media and wells were assessed for CPE daily for 1 week. Samples positive during viral isolation were assessed for viral concentration using a TCID_50_ assay in MARC-145 cells seeded in 96-well plates. Wells were challenged in quadruplicate using 10-fold dilutions of cell supernatants from test wells. After 2 h, fresh media was applied to wells and CPE was assessed daily for 1 week. The 50% endpoint titer was calculated using the method of Reed and Muench (34). TCID_50_ result was converted to log_10_ for further analysis. Viral load in the supernatant from test wells was also assessed using RT-qPCR. RNA was extracted using the MagMAX Pathogen RNA/DNA Kit (Thermo Fisher Scientific) following the manufacturer’s recommendations and stored at −80 °C until analysis. RT-qPCR evaluating the *nsp9* gene was completed as previously described using forward (5′-CCTGCAATTGTCCGCTGGTTTG-3′) and reverse (5′-GACGACAGGCCACCTCTCTTAG-3′) primers, Fluorescein (6-FAM) fluorophore containing nsp9-probe (5′-ACTGCTGCCACGACTTACTGGTCACGCAGT-3′), and Iowa Black quencher (Integrated DNA Technologies) (35). Cycle conditions were as follows: 50 °C for 20 min, 95 °C for 1 min, and 40 cycles of 95 °C for 10 s and 60 °C for 1 min. Quantification was done using 10-fold dilution of a standard stock quantified at 10^11^ copies/μL by OligoCalc (35).

### 2.7 Assessment of IFN stimulated genes

RNA was extracted from cell supernatants using the MagMAX Pathogen RNA/DNA Kit. Complementary DNA was synthesized using random hexamers according to the manufacturer’s recommendations (Invitrogen). Transcription of IFN stimulated genes was assessed using previously described primer sets (Table 1) and SYBR Green-based chemistry (Applied Biosystems) as previously described (36, 37). Briefly, master mix, primers, and template were mixed in a 20 μL reaction volume and reaction conditions were as follows: 95 °C for 15 min, 45 cycles of 95 °C for 15 s and 50 °C for 1 min, and a final dissociation step. Samples were run in duplicate. Transcribed mRNA levels were normalized to β-actin and expressed relative to control using the threshold cycle 2^-∆∆Ct^ calculation method.

**Table 1:** Forward and reverse primers detecting IFN stimulated genes.

Primer	Sequence	Reference
β-actin forward	5′-CTCCTTCCTGGGCATGGA-3′	(36)
β-actin reverse	5′-CGCACTTCATGATCGAGTTGA-3′	(36)
IFN-α forward	5′-GCCTCCTGCACCAGTTCTACA-3′	(36)
IFN-α reverse	5′-TGCATGACACAGGCTTCCA-3′	(36)
IFN-β forward	5′-TGCAACCACCACAATTCC-3′	(36)
IFN-β reverse	5′-CTGAGAATGCCGAAGATCTG-3′	(36)
MCP-1 forward	5′-GCTGATGAGCTACAGAAGAG-3′	(37)
MCP-1 reverse	5′-GCGATGGTCTTGAAGATCAC-3′	(37)
Mx-1 forward	5′-TAGGCAATCAGCCATACG-3′	(36)
Mx-1 reverse	5′-GTTGATGGTCTCCTGCTTAC-3′	(36)
PKR forward	5′-GAGAAGGTAGAGCGTGAAG-3′	(36)
PKR reverse	5′-CCAGCAACCGTAGTAGAG-3′	(36)
TNF-α forward	5′-CCACGTTGTAGCCAATGTC-3′	(36)
TNF-α reverse	5′-CTGGGAGTAGATGAGGTACAG-3′	(36)

### 2.8 Statistical analysis

Statistical analysis was completed in GraphPad Prism version 10.4.2 (GraphPad Software). A one-way ANOVA with a Tukey correction for multiple comparisons was used to compare treatment with different doses of pIFNα and pIFNλ to untreated wells at 24 and 48 hpi. Comparisons between pIFN treatments for the different isolates was done with a two-way ANOVA using a Tukey correction for multiple comparisons. Differences were considered statistically significant when *p* < 0.05. For data generated from single experiments, no statistical testing was performed, and all data discussions are descriptive.

## 3 Results

### 3.1 Evaluation of the efficacy of pIFNα and pIFNλ1 treatment in preventing PRRSV replication in PAMs

**Figure 1:**
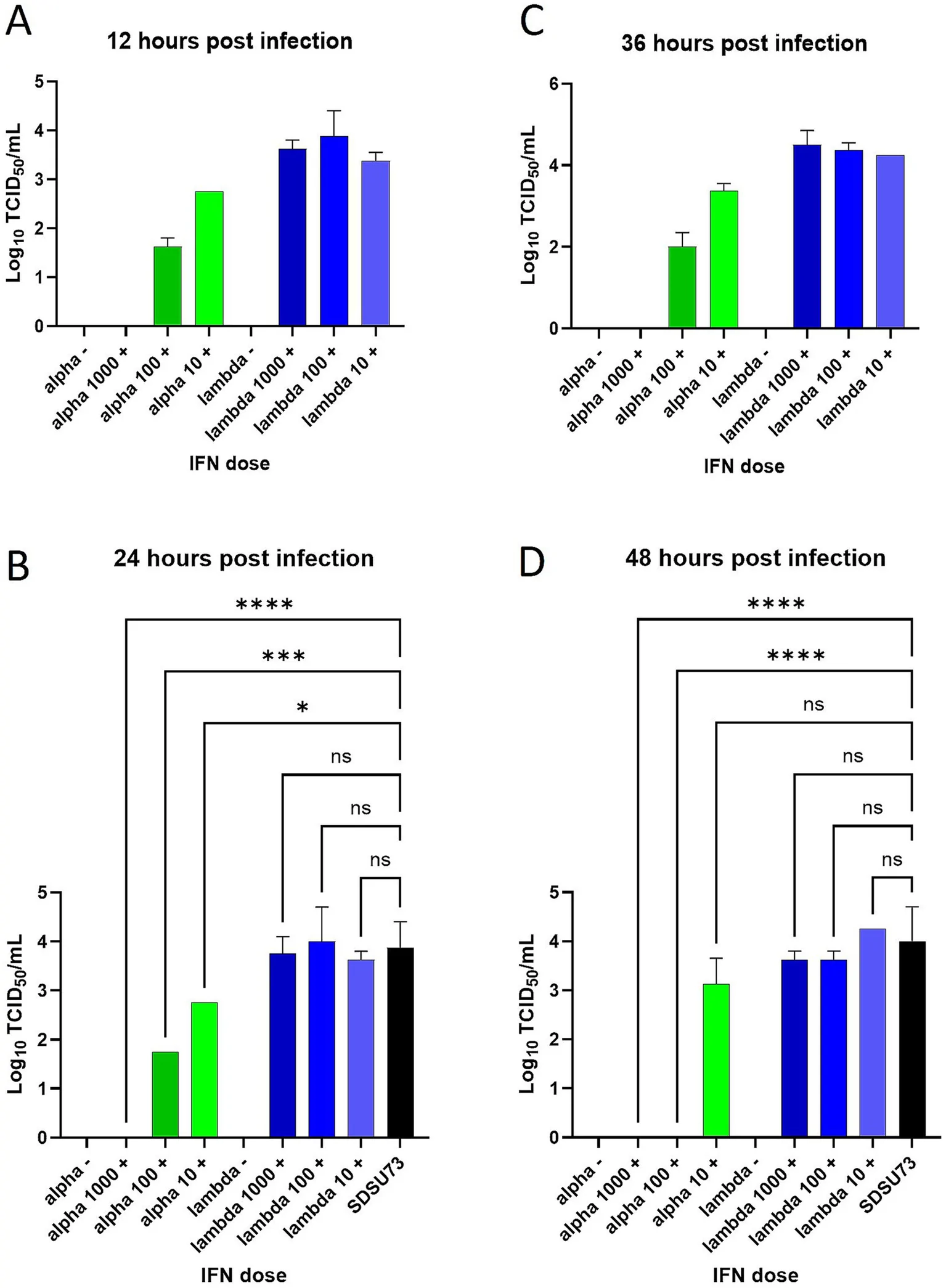
Evaluation of the efficacy pIFN*α* and pIFNλ1 treatment in preventing PRRSV replication in PAMs. PRRSV replication was quantified at 12 **(A)**, 24 **(B)**, 36 **(C)**, and 48 **(D)** hpi *via* TCID_50_ assay and reported as log_10_ TCID_50_/mL. PRRSV challenged cells (+) and unchallenged cells (−) are presented. Data represents two biological replicates using PAMs from different animals. The effect of pIFN treatment was compared to untreated wells at 24 and 48 h using a one-way ANOVA with a Tukey correction for multiple comparisons. Statistical differences at *p* < 0.05 (*), *p* < 0.01 (**), *p* < 0.001 (***), and *p* < 0.0001 (****) are indicated. pIFNα inhibited PRRSV replication in PAMs in a dose-dependent manner. A dose dependent inhibition of replication was not noted with pIFNλ1 treatment. No PRRSV replication was noted in PAMs pretreated with pIFNα at 1000 ng/mL at 12, 24, 36, or 48 hpi.

Recombinant pIFNα and pIFNλ1 were evaluated for their capacity to reduce the replication of PRRSV in PAMs and pIFN titrations were examined to determine an effective dose for further comparisons. pIFNα suppressed the replication of the PRRSV strain SDSU73 in a dose dependent manner with the 1,000 ng/mL treatment preventing replication of PRRSV at 12, 24, 36, and 48 hpi (Figure 1 and Supplementary Table S2). pIFNλ1 did not show a dose dependent effect on PRRSV replication, with no difference in TCID_50_/mL for cells treated with pIFNλ1 at any dose or any time point. At 24 and 48 hpi, PRRSV replication in pIFNα and pIFNλ1 treated cells was compared to PRRSV replication in non-IFN treated PAMs. Cell treated with 1,000 ng/mL or 100 ng/mL of pIFNα were less permissive to SDSU73 replication than untreated cells (*p* < 0.01). No difference in replication was observed between non-IFN treated PAMs and PAMs treated with 10 ng/mL of pIFNα or any dose of pIFNλ1. Follow up evaluations utilized 1,000 ng/mL IFN doses as this was the lowest effective dose that prevented PRRSV replication in PAMs at 12, 24, 36, and 48 hpi.

### 3.2 Efficacy of pIFNα and pIFNλ1 against infection with diverse PRRSV isolates in PAMs

**Figure 2:**
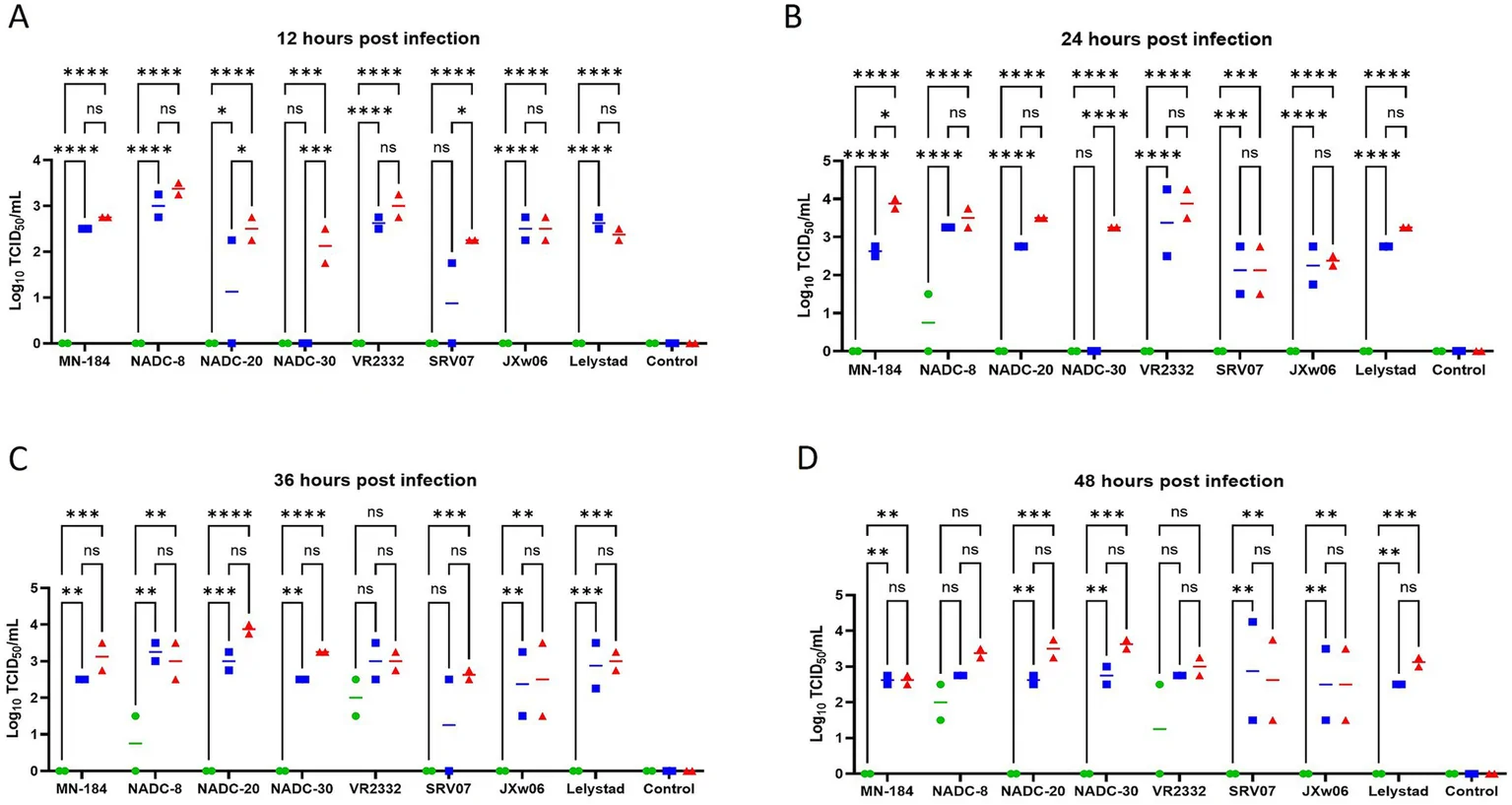
Evaluation of the efficacy pIFNα and pIFNλ1 in preventing the replication of diverse isolates of PRRSV. PRRSV replication was quantified at 12 **(A)**, 24 **(B)**, 36 **(C)**, and 48 **(D)** hpi *via* TCID_50_ assay and reported as log_10_ TCID_50_/mL. Data represents two biological replicates using PAMs from different animals. For each isolate, data were compared using a two-way ANOVA with a Tukey correction for multiple comparisons. Statistical differences at *p* < 0.05 (*), *p* < 0.01 (**), *p* < 0.001 (***), and *p* < 0.0001 (****) are indicated. pIFNα treated PAMs are represented as green circles, pIFNλ1 treated PAMs are represented as blue squares, and non-treated PAMs are represented as red triangles. pIFNα was better able to inhibit PRRSV replication for all PRRSV isolates tested.

To understand the field applicability of pIFN treatment, recombinant IFNs were evaluated for their potential to inhibit the replication of a diverse group of PRRSV isolates containing both type 1 (Lelystad) and 2 (MN-184, NADC-8, NADC-20, NADC-30, VR2332) isolates and two high pathogenicity isolates (SRV07, JXw06) in PAMs using a TCID_50_ assay. The effects of PAM treatment with pIFNα and pIFNλ1 on PRRSV replication was strain dependent (Figure 2 and Supplementary Table S2). For pIFNα treatment, initial inhibition of replication was noted for NADC-8 and VR2332; however, later time points saw no difference between pIFNα treatment and control wells for NADC-8 or VR2332 (48 hpi and 36 and 48 hpi, respectively). pIFNα was better able to inhibit PRRSV replication than pIFNλ1 for all isolates at most time points. Treatment of PAMs with pIFNλ1 had minimal inhibitory effect on PRRSV replication for most strains, especially at the 36 and 48 h time points. pIFNλ1 treatment had the largest impact on the replication of NADC-30, though this effect was statistically significant only at the 12 and 24 hpi time points.

The inhibition of replication was also assessed by quantifying viral RNA for a subset of strains (NADC-8, NADC-20, VR2332, SRV07, Jxw06) (Supplementary Figure S1, Supplementary Table S3). Though none of the differences met the statistical threshold, at 24, 36, and 48 hpi there was a trend indicating increasing concentrations of viral RNA in non-treated PAMs and PAMs treated with pIFNλ1, with pIFNλ1 treated PAMs often falling between pIFNα treated and non-treated PAMs.

### 3.3 Efficacy of pIFNα and pIFNλ1 against PRRSV infection in MARC-145 cells

**Figure 3:**
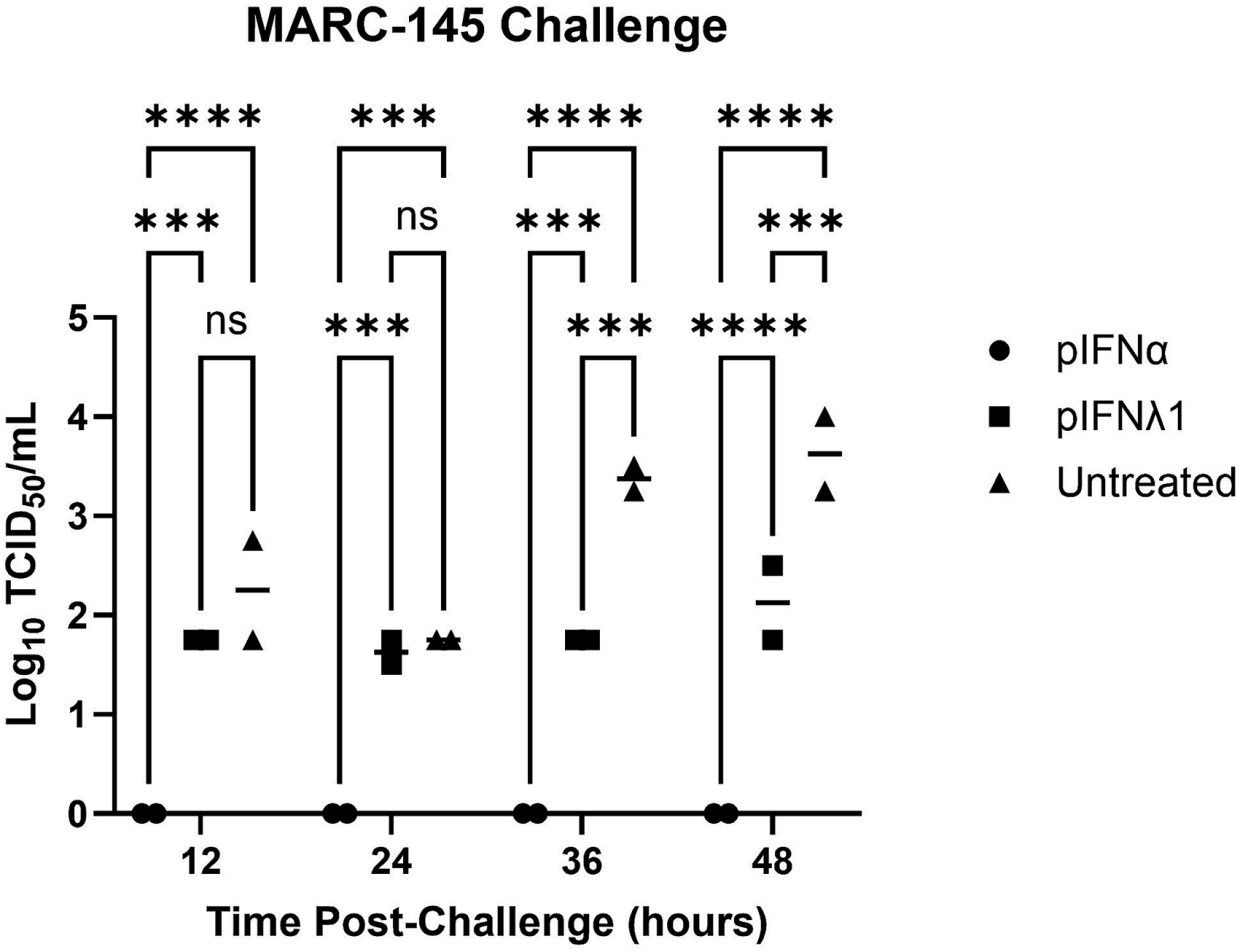
Evaluation of the efficacy pIFNα and pIFNλ1 treatment in preventing PRRSV replication in MARC-145 cells. PRRSV replication was quantified at 12, 24, 36, and 48 hpi *via* TCID_50_ assay and reported as log_10_ TCID_50_/mL. Data represents two technical replicates. Data for each time point were compared using a two-way ANOVA with a Tukey correction for multiple comparisons. Statistical differences at *p* < 0.05 (*), *p* < 0.01 (**), *p* < 0.001 (***), and *p* < 0.0001 (****) are indicated. pIFNα was significantly better than pIFNλ1 at inhibiting PRRSV replication in MARC-145 cells at all time points post challenge. At 36 hpi a difference in PRRSV replication was noted between untreated and pIFNλ1 treated MARC-145 cells.

To better understand how IFN cellular tropism may impact inhibition of viral replication, pIFNα and pIFNλ1 were tested in MARC-145 cells. MARC-145 cells are PRRSV permissive but derived from African green monkey kidney cells, an epithelial derived cell line which may show more responsiveness to pIFNλ1. MARC-145 cells were treated with 1,000 ng/mL of pIFNα or pIFNλ1 or untreated and challenged with SDSU73, a type 2 PRRSV strain. pIFNα was able to inhibit SDSU73 replication at all time points in MARC-145 cells (Figure 3 and Supplementary Table S2). pIFNλ1 treated MARC-145 cells had significantly less PRRSV replication at 36 hpi than the untreated cells; however, there were no differences in replication at 12, 24, or 48 hpi.

### 3.4 Assessment of induction of IFN stimulated genes in PAMs and PK-15 cells

**Figure 4:**
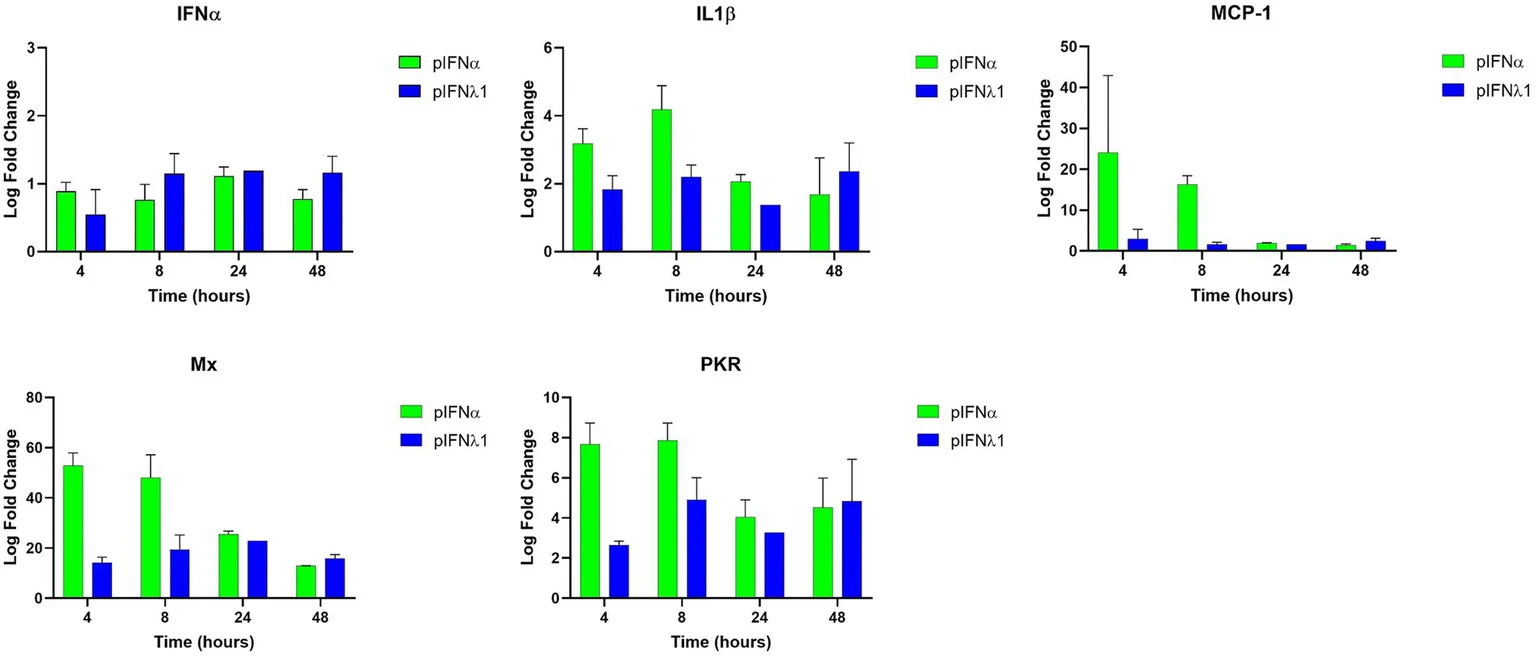
IFN stimulated gene activation following pIFNα and pIFNλ1 treatment in PAMs. Assessment of log fold change of IFN stimulated genes following pIFNα and pIFNλ1 treatment in PAMs. Data represents two biological replicates using PAMs from different animals. Log fold change in transcription at each time point was compared between treatments using a two-way ANOVA with a Tukey correction for multiple comparisons. Changes in IFNα, IL1β, MCP-1, Mx, and PKR were evaluated at 4, 8, 24, and 48 h. pIFNα treatment produced numerically greater transcription of IL1β and MCP-1 and statistically higher Mx, and PKR at early time points, with more comparable changes at 24 and 48 h between treatment with pIFNα and pIFNλ1. Statistical differences at *p* < 0.05 (*), *p* < 0.01 (**), *p* < 0.001 (***), and *p* < 0.0001 (****) are indicated.

To evaluate the cellular effects of recombinant pIFNs on PAMs and PK-15 cells, the change in transcription of IFN stimulated genes (IFNα, IL1β, MCP-1, Mx, and PKR) was evaluated at 4, 8, 12, and 24 h after mock-challenge. PK-15 cells were included to evaluate whether epitheliotropic IFNλ showed increased activity in an epithelial cell line. pIFNα treatment of PAMs induced significant increases in transcription of IL1β, Mx, and PKR at all time points and increases in MCP-1 at 4, 8, and 24 h (Figure 4). pIFNλ1 also stimulated increased transcription of MCP-1, Mx, and PKR at all time points; however greater transcription of MCP-1, Mx, and PKR were noted at 4 and 8 h for PAMs stimulated with pIFNα as compared with pIFNλ1. Transcription of IFN stimulated genes decreased over time for pIFNα stimulation, while pIFNλ1 appeared to have a more sustained activation, making transcription levels for MCP-1, Mx, and PKR comparable at 24 and 48 h between treatments. Comparison of transcriptional levels in PK-15 cells revealed similar phenomenon (Supplementary Figure S2). Transcription of IL1β, Mx, and PKR were higher following stimulation with either pIFNα or pIFNλ1 than that seen in PAMs; however, more responsiveness was seen in PK-15 cells stimulated with pIFNα than pIFNλ1.

### 3.5 Assessment of PEGylated IFNα constructs in MARC-145 cells

**Figure 5:**
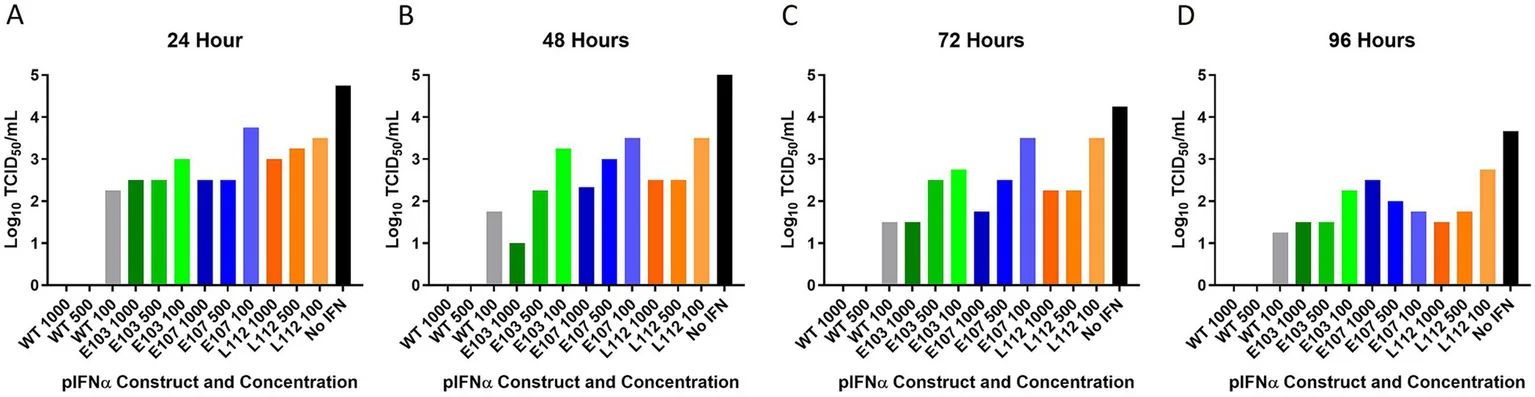
Assessment of pIFNα PEGylated constructs in preventing PRRSV replication. PEGylated pIFNα constructs were assessed for efficacy in preventing PRRSV replication *via* TCID_50_ assay and are presented as log_10_ TCID_50_/mL. Replication was assessed at 24 **(A)**, 48 **(B)**, 72 **(C)**, and 96 **(D)** hpi. PEGylated pIFNα was less effective at preventing PRRSV replication than wild type (WT) pIFNα; however, all PEGylated constructs were able to reduce PRRSV replication at high concentrations. Small differences in efficacy were noted between constructs.

To assess the impact of PEGylation on pIFNα efficacy, three PEGylated IFNα constructs (E103, E107, L112) were tested for efficacy in preventing replication of SDSU73 in MARC-145 cells *via* TCID_50_ assay. The PEGylated IFNα constructs showed reduced efficacy preventing PRRSV replication when compared with the wild type (WT) IFNα at all time points (Figure 5); however, treating MARC-145 cells with 1,000 ng/mL of all constructs resulted in reduced PRRSV replication. The constructs showed minor variation in their ability to prevent PRRSV replication. Similar results were seen with RT-qPCR (data not shown).

## 4 Discussion

IFNs are important innate immune compounds that drive the antiviral state in cells and contribute to the adaptive immune response to intracellular pathogens (1–3). IFNs have been of interest to the swine industry for control of PRRSV, an economically devastating agent resulting in estimated industry losses of $1.2 billion annually (6). IFNs have been assessed *in vitro* for their capacity to limit PRRSV replication in MARC-145 cells (10, 12, 13, 19, 20), a PRRSV permissive cell line derived from monkey kidney cells and in primary alveolar macrophages and monocytes (7, 11, 16–18, 38). IFNs have also be assessed *in vivo* as therapeutics to limit disease severity or modulate the adaptive immune response (13–15). Here, we evaluated the ability of recombinant porcine IFN-α and IFN-λ to prevent PRRSV replication in porcine PAMs, the primary cell type infected by PRRSV. More importantly, this investigation assessed the ability of IFNs to limit replication across a genotypically diverse subset of PRRSV isolates and also investigated the impact of PEGylation on pIFNα activity.

Our initial assessments evaluated the dose responsiveness of the PRRSV strain SDSU73 to pIFNα and pIFNλ1. Similar to previous observations with other PRRSV isolates, SDSU73 showed a dose dependent inhibition of replication in response to IFNα treatment (13), with complete inhibition of replication observed only with PAMs treated at 1000 ng/mL (Figure 1). In contrast to previous research indicating a dose-dependent response to IFNλ treatment, we saw no impact on PRRSV replication following pIFNλ1 treatment (12, 17). The differences between our results and prior work may be due to differences in strain susceptibility to IFNλ, as we have shown in this study that different PRRSV strains have different responsiveness to recombinant pIFN (Figure 2). Differences could also be associated with the IFNλ source and subtype (human versus swine), cells used (MARC145 versus PAMs), or the assay used to quantify PRRSV replication (plaque forming units versus TCID_50_) (12). Although prior work indicated IFNλ treatment reduced viral replication, it was not able to completely eliminate replication (12, 17), as was seen with higher doses of IFNα in this study. Our results also confirm increased PRRSV sensitivity to IFNα compared with IFNλ (12).

To further assess the utility of pIFNα and pIFNλ1 in limiting PRRSV replication, we evaluated replication of a genetically diverse subset of isolates. Similar to previous work and the results of initial trials with SDSU73 (12), pIFNα was better able to inhibit the replication of most PRRSV isolates than pIFNλ1. While pIFNα showed good efficacy against most of the tested PRRSV strains, two strains (NADC-8 and VR2332) exhibited reduced susceptibility to pIFNα treatment and were able to replicate at later time points post-infection (36–48 hpi). Genetic comparison of the ORF5 gene revealed that NADC-8 and VR2332 had higher sequence identity with each other than any other strain tested (data not shown) and they are both PRRSV-2 lineage 5 viruses, indicating replication in the face of pIFNα may be an attribute of these more genetically similar viruses. Differences in pIFNα susceptibility have been seen previously using field strains (18), although our analysis included a more diverse subset of strains. We also noted differences in susceptibility to pIFNλ1 between strains, with NADC-30 showing the greatest susceptibility; however, reduced replication was only noted at early time points (12–24 hpi). Interestingly, we saw differences in replication when evaluating TCID_50_ analysis; however, there were no statistical differences between treatments noted when assessing RT-qPCR results. This may be associated with reduced viral protein translation or viral particle assembly in pIFNα treated cells without concomitant reductions in viral genome replication.

We also evaluated gene transcription of IFN-stimulated genes in PAMs to evaluate the responsiveness of PAMs to recombinant IFN treatment. pIFNα stimulated larger increases in transcription in IL1β, MCP-1, Mx, and PKR than pIFNλ1. This may provide some insight into the differences seen in PRRSV replication between IFN treatments, with inhibition of replication being associated with an elevated antiviral state in PAMs pretreated with pIFNα. Our results contrast with previous work evaluating IFNλ, which showed IFNλ induces the MxA gene at higher levels than IFNα in MARC-145 cells (12); however, differences in IFN responses between PAMs and MARC-145 cells have been described (39).

Because the IFNλ receptor (IL28RA) has been previously shown to have a restricted expression pattern and stimulate greater effects in epithelial cell types (40–42), we also assessed the impact of recombinant IFNs on transcription of ISGs in PK-15 cells, a porcine kidney cell line, and the effects of recombinant IFN treatment on PRRSV replication using MARC-145 cells, a PRRSV permissive cell line derived from epithelial origin. Although epithelial cell lines were expected to be more responsive to pIFNλ1 treatment and transcription of some IFN-stimulated genes was higher for PK-15 cells when compared with PAMs, the response of PK-15 cells was much greater to pIFNα stimulation. Additionally, pIFNα was able to inhibit PRRSV replication in MARC-145 cells, while pIFNλ1 did not eliminate replication. The broader cellular response and better replication inhibition seen with pIFNα indicates pIFNα is a better biotherapeutic candidate for PRRSV and may be more efficacious for epitheliotropic viruses as well.

PEGylation of cytokines can extend the half-life allowing for a longer duration of effect and enable extended dosing intervals; however, it also causes reductions in the biological effect (21). This can be overcome by the extended duration of effect, making PEGylation a viable mechanism to increase the practicality of biotherapeutic agents. The final assessment in this study was an evaluation of the impact of PEGylation on the capacity of pIFNα to inhibit PRRSV replication in MARC-145 cells. As expected, a decline in pIFNα efficacy was noted after PEGylation; however, replication was limited by treatment with higher doses of the PEGylated pIFNα constructs (greater than 500 ng/mL). Similar reductions in efficacy of IFN have been seen previously following PEGylation without precluding their clinical use (43, 44).

This report is the first to assess the effects of pIFNα and pIFNλ1 on a genetically diverse group of PRRSV isolates. pIFNα was better able to inhibit replication of the evaluated PRRSV isolates and induce IFN-stimulated gene transcription, making it a better candidate for PRRSV intervention. Evaluation of pIFNα PEGylated constructs indicated good efficacy limiting PRRSV replication at higher concentrations *in vitro*, indicating constructs have potential in controlling PRRSV replication *in vivo* and further pharmacokinetic and efficacy testing is warranted.

## Data Availability

The raw data supporting the conclusions of this article will be made available by the authors, without undue reservation.

## Supplementary material

SUPPLEMENTARY FIGURE S1 Evaluation of the efficacy pIFNα and pIFNλ1 in preventing the replication of diverse isolates of PRRSV *via* RT-qPCR. PRRSV replication was quantified at 12 **(A)**, 24 **(B)**, 36 **(C)**, and 48 **(D)** hpi *via* RT-qPCR and reported as log_10_ copy number/mL. pIFNα treated PAMs are represented as circles, pIFNλ1 treated PAMs are represented as squares, and non-treated PAMs are represented as triangles. Data represents two biological replicates using PAMs from different pigs. For each isolate, data were compared using a two-way ANOVA with a Tukey correction for multiple comparisons. No statistical differences in viral RNA were detected but the viral copy number tended to rise in pIFNλ1 and untreated PAMs and remain consistent in pIFNα treated PAMs over the study period.

SUPPLEMENTARY FIGURE S2 IFN stimulated gene activation following pIFNα and pIFNλ1 treatment in PK-15 cells. Assessment of log fold change of IFN stimulated genes following pIFNα and pIFNλ1 treatment in PK-15 cells. Changes in IFNα, IL1β, MCP-1, Mx, and PKR were evaluated at 4, 8, 24, and 48 h. Both pIFNα and pIFNλ1 stimulation caused increases in IFN stimulated gene production; however, pIFNα caused greater transcription of IL1β, MCP-1, Mx, and PKR at all time points.
